# Molecular Insight into TdfH-Mediated Zinc Piracy from Human Calprotectin by Neisseria gonorrhoeae

**DOI:** 10.1128/mBio.00949-20

**Published:** 2020-05-26

**Authors:** Michael T. Kammerman, Aloke Bera, Runrun Wu, Simone A. Harrison, C. Noel Maxwell, Karl Lundquist, Nicholas Noinaj, Walter J. Chazin, Cynthia Nau Cornelissen

**Affiliations:** a Center for Translational Immunology, Institute for Biomedical Sciences, Georgia State University, Atlanta, Georgia, USA; b Markey Center for Structural Biology, Department of Biological Science, Purdue University, West Lafayette, Indiana, USA; c Purdue Institute of Inflammation, Immunology and Infectious Disease, Purdue University, West Lafayette, Indiana, USA; d Department of Biological Sciences, Purdue University, West Lafayette, Indiana, USA; e Department of Biochemistry, Vanderbilt University, Nashville, Tennessee, USA; f Department of Chemistry, Vanderbilt University, Nashville, Tennessee, USA; g Center for Structural Biology, Vanderbilt University, Nashville, Tennessee, USA; University of Illinois at Chicago

**Keywords:** isothermal calorimetry, calprotectin, protein-protein interactions, *N. gonorrhoeae*, *Neisseria gonorrhoeae*, TonB-dependent transporter

## Abstract

Neisseria gonorrhoeae, responsible for the sexually transmitted infection gonorrhea, is an obligate human pathogen exquisitely adapted for survival on mucosal surfaces of humans. This host-pathogen relationship has resulted in evolution by N. gonorrhoeae of pathways that enable the use of host metalloproteins as required nutrients through the deployment of outer membrane-bound TonB-dependent transporters (TdTs). Recently, a TdT called TdfH was implicated in binding to calprotectin (CP) and in removal of the bound zinc (Zn), enabling gonococcal growth. TdfH is highly conserved among the pathogenic *Neisseria* species, making it a potentially promising candidate for inclusion into a gonococcal vaccine. Currently, the nature and specificity of the TdfH-CP interaction have not been determined. In this study, we found that TdfH specifically interacted with human calprotectin (hCP) and that growth of the gonococcus was supported in a TdfH-dependent manner only when hCP was available as a sole zinc source and not when mouse CP was provided. The binding interactions between TdfH and hCP were assessed using isothermal titration calorimetry where we observed a multistate model having both high-affinity and low-affinity sites of interaction. hCP has two Zn binding sites, and gonococcal growth assays using hCP mutants deficient in one or both of the Zn binding sites revealed that TdfH exhibited a site preference during Zn piracy and utilization. This report provides the first insights into the molecular mechanism of Zn piracy by neisserial TdfH and further highlights the obligate human nature of N. gonorrhoeae and the high-affinity interactions occurring between TdTs and their human ligands during pathogenesis.

## Abstract (precis)

The dramatic rise in antimicrobial resistance among Neisseria gonorrhoeae isolates over the last few decades, paired with dwindling treatment options and the lack of a protective vaccine, has prompted increased interest in identifying new bacterial targets for the treatment and, ideally, prevention of gonococcal disease. TonB-dependent transporters are a conserved set of proteins that serve crucial functions for bacterial survival within the host. In this study, binding between the gonococcal transporter, TdfH, and calprotectin was determined to be of high affinity and host restricted. The current study identified a preferential TdfH interaction at the calprotectin dimer interface. An antigonococcal therapeutic could potentially block this site on calprotectin, interrupting Zn uptake by N. gonorrhoeae and thereby prohibiting continued bacterial growth. We describe protein-protein interactions between TdfH and calprotectin, and our findings provide the building blocks for future therapeutic or prophylactic targets.

## INTRODUCTION

Neisseria gonorrhoeae is responsible for the sexually transmitted infection gonorrhea and has shown a steady rise in infections worldwide over the last decade (1, 2). In 2018 alone, the number of reported gonococcal infections reached over 500,000 in the United States and over 87 million worldwide (1, 3). Increasing antimicrobial resistance among recently isolated strains has complicated the treatment of this infection (4, 5). The accumulation of antimicrobial resistance has left clinicians with few remaining therapies. The current CDC-recommended treatment is dual therapy with ceftriaxone plus azithromycin (4). A recent case study in the United Kingdom reported a patient infected by a gonococcal strain exhibiting high levels of resistance to both drugs in the dual therapy, marking the beginning of an era where there may be no effective treatments for gonococcal infections (6, 7). The lack of protective immunity against N. gonorrhoeae after infections (8, 9), coupled with the closing window of treatments available, highlights the need for new therapeutics or, ideally, vaccine interventions that would prevent gonococcal diseases.

In order to inhibit microbial invaders from multiplying, mammalian hosts deploy “nutritional immunity” as a means to restrict availability of essential trace metals through the action of metal-binding proteins (10). This protective mechanism was first described in the context of iron deprivation but extends to other transition metals as well (10, 11); metal sequestration in combination with tight control of metal metabolism is used to deplete sites of infection of free metals. N. gonorrhoeae is highly effective at subverting host nutritional immunity by hijacking human metal-binding proteins and using the metal cargo for growth and survival (10, 12–16). This “metal piracy” is accomplished via a family of outer membrane transporters, known as TonB-dependent transporters (TdTs). These transporters depend on the TonB-ExbB-ExbD complex of proteins to harness the energy generated by the proton motive force across the inner membrane (17, 18). The gonococcus can utilize iron bound to human transferrin and lactoferrin and has recently been shown to utilize S100A7 for Zn-dependent growth (16, 19, 20).

The gonococcal genome encodes eight known TdTs, with five of these transporters binding to a known host ligand (17, 21). Iron acquisition via transferrin is accomplished through TbpA, which demonstrates species specificity for only human transferrin (19, 22). Similarly, Zn acquisition from S100A7 is achieved via the production of the gonococcal transporter TdfJ and exhibits a similar species restriction for ligand binding (20). N. gonorrhoeae has also been shown to utilize calprotectin (CP; a heterodimer of S100A8 and S100A9) for survival in neutrophil extracellular traps [NETs]) (23). Zn piracy from CP has been described as being TdfH dependent; however, the species specificity of this interaction is not known (23, 24).

TdfH is highly conserved among the pathogenic *Neisseria* species, making it a promising candidate for vaccine or drug design (24). Insight gained in this study provide a blueprint for further investigations of the vaccine potential of TdfH and new therapeutics that disrupt the TdfH-hCP interaction.

## RESULTS

### N. gonorrhoeae growth is not supported by mCP and shows preferential binding of hCP.

**FIG 1:**
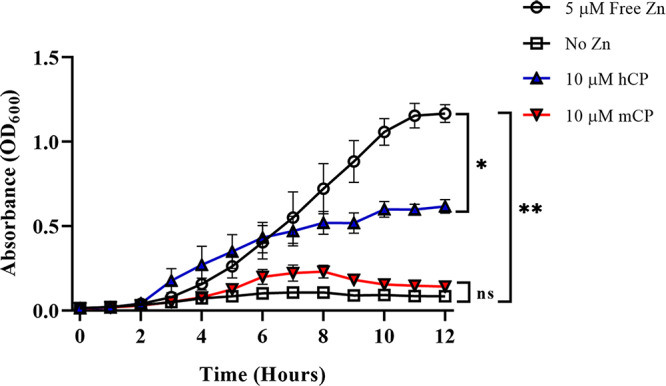
Growth of Neisseria gonorrhoeae when mCP is supplied as the sole Zn source. Gonococcal cells were allowed to double in CDM and were then diluted to an OD_600_ of ∼0.02 and transferred to a 96-well microtiter plate containing concentrated growth premixes. Cells supplemented with mCP as the sole Zn source (red inverted triangles) were significantly deficient in their ability to support growth of the gonococcus beginning at 6 h (*, *P < *0.05; **, *P < *0.01) compared to the free-Zn positive control (black open circles) and WT CP (blue triangles). There was no statistically significant difference (ns) in growth between the cells receiving the no-Zn treatment and cells supplemented with mCP as the sole Zn source. Significance was determined via a two-way ANOVA with Tukey posttest. Error bars represent standard errors of the means (SEM) of results from three independent experiments performed in technical triplicate.

Jean et al. (23) demonstrated that the gonococcus was able to use CP in a TdfH-dependent fashion, resulting in Zn accumulation. Furthermore, this study demonstrated an *in vivo* relevance of the production of TdfH in that production of this transporter enabled the gonococcus to better survive killing by neutrophil extracellular traps (NETs) (23). While Jean et al. demonstrated a direct interaction between CP and whole, TdfH-producing gonococcal cells, the molecular basis of the interaction was not determined nor was the affinity measured (22). In the current study, we first investigated whether the interactions between TdfH and CP were species specific. Previously studied gonococcal TdTs bind and acquire metals specifically from the human forms of their ligands (20, 22, 25). To test whether this was also true for TdfH, gonococcal cells were grown in Chelex defined media (CDM), which is a metal-depleted medium, that was supplemented with either 25% saturated mouse calprotectin (mCP) or human calprotectin (hCP) as the sole Zn source. Cells that were grown with 25% saturated mCP demonstrated significantly impaired growth, compared to cultures grown with 5 μM free Zn and 10 μM hCP (Fig. 1).

**FIG 2:**
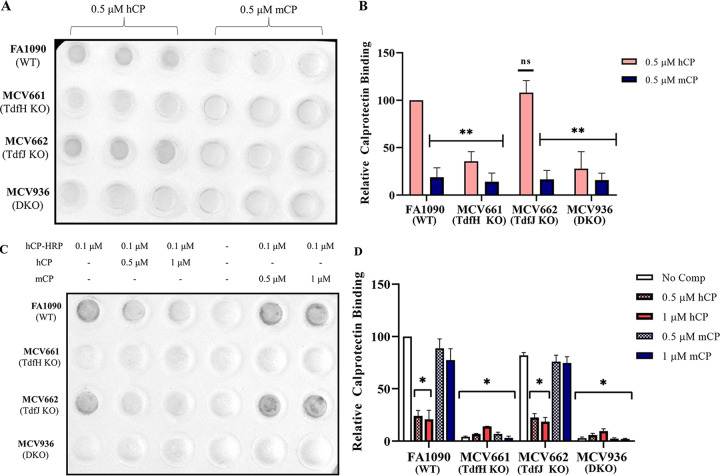
hCP and mCP competition dot blot assay. (A) Representative image of direct CP binding assays. N. gonorrhoeae strains FA1090 (WT), MCV661 (TdfH KO), MCV662 (TdfJ KO), and MCV936 (DKO) were grown under Zn-restricted conditions and applied to nitrocellulose membrane at a standardized density. Blots were probed with 0.5 μM hCP or 0.5 μM mCP. CP bound to the surface of cells was detected with an anti-S100A9 monoclonal antibody followed by detection using an anti-mouse IgG conjugated to HRP. (B) Densitometry analysis of results from three independent biological replicates performed in technical triplicate. Densitometry analysis of scanned blots demonstrated a significant reduction in the binding of mCP to affixed gonococci compared to the binding of hCP to the WT strain. (C) Representative image of competitive CP binding assays performed with N. gonorrhoeae strains FA1090 (WT), MCV661 (TdfH KO), MCV662 (TdfJ KO), and MCV936 (DKO). Membranes were probed with either 0.1 μM hCP-HRP alone or a mixture of 0.1 μM hCP-HRP with 0.5 μM or 10 μM hCP or mCP unlabeled competitor. (D) Densitometry analysis of biological triplicate sets of dot blots. Densitometry analysis was accomplished using Bio-Rad’s Image Lab software. Significance was calculated using unpaired Student’s *t* tests with comparisons made to FA1090 probed with hCP. Error bars represent SEM (*, *P < *0.05; **, *P < *0.01).

TdfH has been previously reported to be necessary for binding to hCP in whole gonococcal cells (23). In an attempt to further define the species specificity of the interaction between TdfH and CP, dot blots were probed with hCP and mCP directly or in competition with each other. Whole cells of the following strains were immobilized onto a nitrocellulose membrane: FA1090 (wild type [WT]), MCV661 (TdfH knockout [KO]), MCV662 (TdfJ KO), and MCV936 (TdfH/TdfJ KO) (as described in Table 1). Membranes were probed with either 0.5 μM hCP or 0.5 μM mCP (Fig. 2A) followed by an anti-S100A9 monoclonal antibody that is cross-reactive for hCP and mCP. The blots were developed with an anti-rabbit IgG-horseradish peroxidase (HRP) secondary conjugate. The WT and TdfJ KO strains, both of which express TdfH, bound hCP but not mCP. Likewise, cells lacking TdfH (TdfH KO and the TdfH and TdfJ double KO [DKO] cells) did not bind either of the calprotectins. Quantitative measures of these blots were determined through densitometry analysis (Fig. 2B), which showed significantly reduced mCP binding (by ∼60% or more) in all strains compared to hCP binding to the WT. Further, all mutant strains except the TdfJ KO showed a reduction of more than 60% in hCP binding. A competition assay was developed to assess whether hCP and mCP would be able to compete for binding to TdfH. Nitrocellulose-bound cells were blocked and probed with hCP conjugated to horseradish peroxidase (hCP-HRP) alone, with hCP-HRP mixed with a 5-fold or 10-fold molar excess of hCP or with hCP-HRP plus a 5-fold or 10-fold molar excess of mCP. The WT strain FA1090 showed a decrease in the HRP signal in the presence of an unlabeled hCP competitor (Fig. 2C, second and third columns). mCP competitor at both concentrations did not reduce the HRP development of the blots (Fig. 2C, last two columns). The second row, which contained TdfH KO strain MCV661, showed background levels of HRP development (Fig. 2C). The third row of the blot contained a TdfJ KO strain and also exhibited a decrease in development when probed with hCP competitor (Fig. 2C, second and third columns) but demonstrated no reduction in development when mCP was used as a competitor (Fig. 2C, last two columns). The fourth and final row of the blot contained TdfH and TdfJ double knockout (DKO) strain MCV936, which exhibited background levels of development when probed with either hCP or mCP competitors (Fig. 2C). Densitometry scans of biological triplicate competition assays were used to quantify the reduction in hCP-HRP binding to the cell surface (Fig. 2D). When blots were probed with either a 5-fold or 10-fold molar excess of hCP competitor, a significant reduction in HRP signal was observed (*P* < 0.05); no significant reduction in signal was seen when mCP was added as a competitor.

**TABLE 1:** Neisserial strains used in this study

Strain	Genotype and or relevant characteristic(s)^*a*^	Reference
FA19	WT	59
FA1090	WT	60
MCV661	FA1090 *tdfH*::Ω (Str^r^, Spr^r^)	61
MCV662	FA1090 *tdfJ*::Ω (Str^r^, Spr^r^)	61
MCV936	FA1090 *tdfH*::Ω *tdfJ*::<Kan2>	23

a Str^r^, streptomycin resistance; Spr^r^, spectinomycin resistance.

### TdfH and human calprotectin form complexes detected by size exclusion chromatography (SEC).

**FIG 3:**
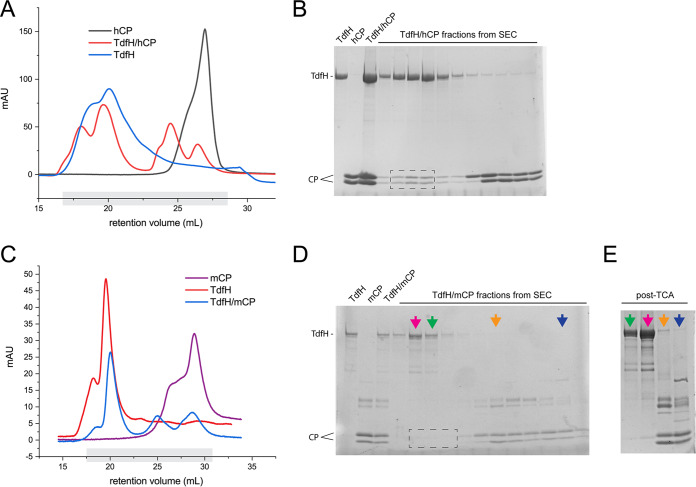
Formation of recombinant TdfH and calprotectin complexes. (A) Recombinant TdfH (blue), hCP (black), or TdfH incubated with hCP (red) was run over a Superdex 200 column. The fractions collected, which are highlighted in gray, were run on a 15% SDS-PAGE gel. mAU, milli-absorbance units. (B) SDS-PAGE (15%) was performed with TdfH alone, hCP alone, or TdfH and mCP incubated together, and fractions were collected. TdfH incubation with hCP resulted in coelution in fractions collected from SEC (dotted box) indicating the formation of a TdfH-hCP complex. (C) Recombinant TdfH (red), mCP (purple), and TdfH incubated with mCP (blue) were run over a Superdex 200 column. Fractions collected for SDS-PAGE analysis are highlighted in gray. (D) SDS-PAGE (15% gel) of TdfH, mCP, and collected fractions of TdfH incubated with mCP. TdfH and mCP that had been incubated together independently eluted during SEC (dotted box). (E) Two fractions that eluted only TdfH (purple and green arrows) and only mCP (orange and blue arrows) were chosen for TCA precipitation to see if any amounts of TdfH and mCP eluted together. The precipitated TCA contained only TdfH (purple and green) or mCP (orange and blue), indicating an inability for TdfH-mCP complexes to form.

To determine whether we could recapitulate the TdfH interaction with hCP *in vitro*, we subjected the gene sequence for TdfH to codon optimization and subcloned the gene into the pHIS2 vector and pET20b vector (modified with an N-terminal 10× His tag and tobacco etch virus [TEV] protease site) for expression in Escherichia coli. While the native expression (pET20b vector) was only barely observable by Western blotting, we were able to express TdfH into inclusion bodies (pHIS2 vector) with high yields. We refolded and purified TdfH using a nickel-nitrilotriacetic acid (Ni-NTA) column and performed a final purification into 1× phosphate-buffered saline (PBS) with 0.05% n-dodecyl-β-d-maltoside (DDM) using size exclusion chromatography. The sample was then mixed using TdfH at a ratio of 1:2 with each hCP and mCP, incubated for at least 1 h, and then separated again using size exclusion chromatography into 1× phosphate-buffered saline (PBS) with 0.05% DDM. Similarly, control samples of hCP and mCP were also analyzed for comparison. As shown in Fig. 3A, we observed a clear shift in hCP such that it coeluted with TdfH, as visualized by SDS-PAGE analysis (Fig. 3B). Conversely, no observable shift was detected with mCP (Fig. 3C and D), even after trichloroacetic acid (TCA) precipitation of the samples to boost the low signal from mCP (Fig. 3E).

### Human calprotectin and TdfH interact with nanomolar affinity.

**FIG 4:**
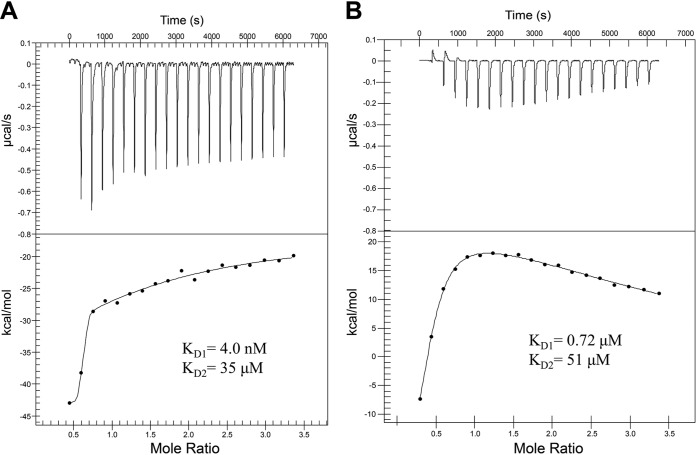
Isothermal titration calorimetry of hCP and mCP with TdfH. Calprotectin was titrated into TdfH over 20 injections. (A) Using NanoAnalyze, the isotherm of the hCP injections best fit a multisite model and resulted in high and low affinities (equilibrium dissociation constant [*K_D_*]) of 4.0 nM and 35 μM. (B) The mCP titration isotherm had dramatically reduced levels of kcal/mol heat release compared to hCP. The mCP isotherm best fits a two-state model with calculated high and low affinities of 0.72 μM and 51 μM.

One of the signature symptoms of gonococcal infection is the influx of neutrophils into the site of infection, driven by localized inflammation (26–28). Human neutrophils can undergo a process of NETosis, releasing their intracellular and granular contents, including the highly abundant cytosolic protein hCP (29–31). hCP has been documented to reach concentrations as high as 1 mg/ml in inflamed tissues. We therefore investigated whether this high-abundance neutrophil protein, found on inflamed mucosal membranes, interacted with TdfH with high affinity. Isothermal titration calorimetry (ITC) was used to determine the binding affinity of the interaction between TdfH and hCP. Here, 300 μl of 20 μM recombinantly produced TdfH was loaded into the sample well of a nano-ITC microcalorimeter, and 2.5 μl of 200 μM hCP was incrementally titrated into the TdfH over 20 injections. Using the NanoAnalyze software package, the isotherm of the TdfH and hCP ITC experiment (Fig. 4A) was found to best fit a two-state model, allowing us to determine the binding parameters for the interaction. Control experiments were conducted using hCP and mCP titrated into the sample cell containing only buffer to demonstrate that these observed binding parameters were not due to changes in the oligomeric state of CP alone. Two distinct binding profiles were observed, indicating multiple modes of interaction in a two-state model. The first was a high-affinity interaction with the affinity calculated to be 4.0 nM (see Table S1 in the supplemental material). The second binding profile was a low-affinity interaction with the affinity calculated to be 35 μM. The lack of growth support, competition, and complex formation with TdfH by mCP lead us to question whether mCP had any detectable interaction with TdfH. Similarly to the ITC of hCP, a titration of mCP was performed and analyzed using a two-state model (Fig. 4B). Similarly to the addition of hCP, the titration of mCP into the TdfH-containing cell yielded two affinities, with the higher affinity determined to be 0.72 μM and the lower 51 μM (Table S1). However, unlike the hCP interaction, there was less overall heat release (kcal/mol) when mCP was added to TdfH.

10.1128/mBio.00949-20.1 TABLE S1 Summary of isothermal titration calorimetry parameters. The table summarizes the results and analysis of the ITC experiments for each of the calprotectin constructs tested with TdfH. The analysis was performed as described in Materials and Methods, reporting here the best-fit model used and the calculated values for *K_a_* (absorption rate constant), *n*, Δ*H*, and Δ*S* for each experiment. Data analysis and error calculations were performed using the NanoAnalyze software package (TA Instruments). Download Table S1, DOCX file, 0.03 MB. Copyright © 2020 Kammerman et al. 2020 Kammerman et al. This content is distributed under the terms of the Creative Commons Attribution 4.0 International license.

### hCP and mCP share limited sequence identity.

**FIG 5:**
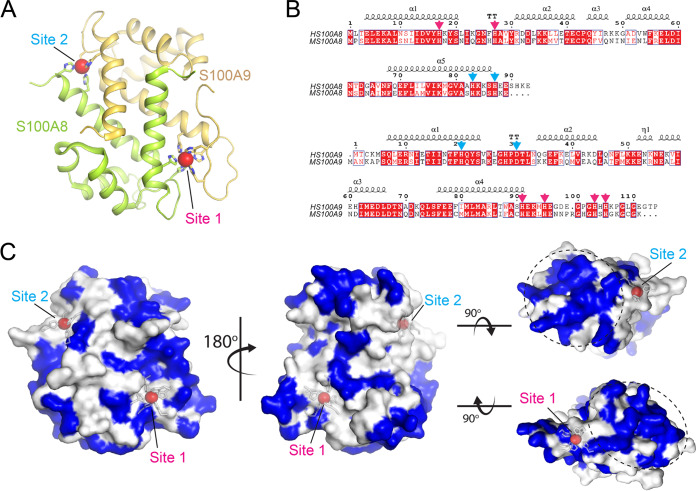
Pairwise alignment of human and mouse S100A8 and S100A9 proteins. (A) The structure of human calprotectin (PDB ID 4GGF) in complex with manganese (red spheres). The side chains of the metal-binding sites are included in stick representation. (B) A sequence alignment of human and mouse S100A8 and S100A9, with mapped secondary structure elements. Sequences for human (AAH05928.1) and mouse (NP_038678.1) S100A8 proteins were aligned through Geneious with a BLOSUM 65 matrix. The S100A8 sequences share 58% identity and 83% sequence similarity. Mouse S100A8 is 89 amino acids in length, whereas human S100A8 is 93 amino acids in length. Sequences for human (AAH47681.1) and mouse (NP_001268781.1) S100A9 proteins were aligned and shared 58% identity and 74% sequence similarity. Mouse S100A9 is 113 residues in length, whereas human S100A9 is 114 residues in length. Residues making up the site 1 and site 2 metal-binding sites are indicated by magenta and cyan arrows, respectively. (C) Based on the sequence alignment in panel B, residues that are different between human and mouse calprotectin are colored blue on the surface of the human calprotectin. The most divergent regions between the two are found on one side of site 1 and on the diametrically opposed side of the site 2 metal-binding site (indicated by the dashed circles).

The ability of TdfH to differentiate between the human and mouse forms of CP led us to investigate the similarity between the amino acid sequences of the proteins. CP is an obligate dimer of S100A8 and S100A9 and contains two transition metal-binding sites termed site 1 (S2) and site 2 (S2) (Fig. 5) (32). The sequences for human S100A8 (GenBank accession no. AAH05928.1) and S100A9 (AAH47681.1) were aligned with mouse S100A8 (NP_038678.1) and S100A9 (NP_001268781.1) using Geneious software and then visualized using ESPript 3 (Fig. 5B) (33, 34). Human and mouse S100A8 shared 58% sequence identity and 83% similarity, while the S100A9 proteins shared 58% sequence identity and 74% similarity. Mapping the divergent residues to the surface of the hCP structure revealed that most of the diversity was to be found at the ends of the CP structure in proximity to the transition metal-binding sites (Fig. 5C).

### S1KO hCP is unable to support the growth of N. gonorrhoeae.

**FIG 6:**
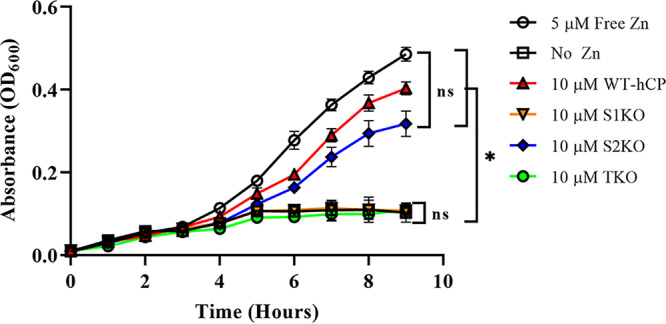
Growth of N. gonorrhoeae when hCP Zn site knockouts are used as the sole Zn source. Gonococcal strain FA19 was grown with concentrated premixes containing hCP and hCP site knockouts as the sole Zn source. hCP was saturated to 25% with ZnSO_4_ and dialyzed overnight against the native buffer containing Chelex-100 resin to remove any unbound residual Zn. FA19 that had been grown with 5 μM free Zn (black open circles), 10 μM WT-CP (red triangles), and 10 μM S2KO (blue diamonds) showed significantly increased growth compared to the no-Zn treatment results (back open boxes) and showed no statistically significant differences in growth compared to each other. The free Zn, WT, and S2KO strains also demonstrated a significant growth increase compared to the S1KO strain (orange inverted triangles) and TKO strain (green circles). The levels of growth of the S1KO and TKO strains were not significantly different from the level of growth of our no-Zn negative-control strain, which displayed minimal growth over the 9-h incubation. Statistical analyses of biological triplicates performed in technical triplicate were done via a two-way ANOVA with a Tukey *post hoc* test. *, *P < *0.05. Error bars represent standard errors of the means (SEM).

The antimicrobial properties of hCP have been found to negatively affect a variety of microorganisms, including Staphylococcus aureus and Candida albicans (35–37). This antimicrobial effect has been attributed to the metal sequestration properties of the protein. hCP has two sites available for metal sequestration. Site 1 is composed of 6× His residues and binds a range of metals with very high affinity, including Zn, copper (Cu), and manganese (Mn). Site 2 is composed of 3 His residues and 1 Asp residue and binds tetravalent metals with high affinity such as Zn and Cu but does not bind Mn (38). To determine whether TdfH was capable of Zn acquisition from both metal-coordinating sites of hCP, a series of FA19 cultures were grown with the following sole Zn sources: WT hCP, site 1 knockout hCP (S1KO), site 2 knockout hCP (S2KO), and a total knockout hCP (TKO), which binds no transition metals with high affinity (Fig. 6). Calprotectin added to the concentrated growth premixes had previously been loaded with ZnSO_4_ to achieve 25% saturation. Cells grown in the presence of S1KO as the sole Zn source demonstrated limited growth, similar to that of the no-Zn negative-control strain. Similarly, gonococci grown in the presence of the TKO grew in a manner that was not significantly different from that seen with the no-Zn control. Gonococci grown with S2KO as the sole Zn source, by contrast, demonstrated growth patterns that were not significantly different from those seen with cells grown using the WT hCP. Free Zn, WT hCP, and the S2KO hCP all supported growth at a level that was significantly higher than that seen with the no-Zn, S1KO, and TKO hCP controls. No significant differences were noted in the growth patterns when gonococci were provided free Zn, WT hCP, or S2KO as the sole Zn source. The lack of growth support detected with the S1KO provides strong evidence that the interaction between TdfH and hCP is located predominantly around site 1 and is potentially the primary location of Zn piracy from hCP by the gonococcus.

### TdfH interaction with hCP depends on the sequence at each Zn binding site.

**FIG 7:**
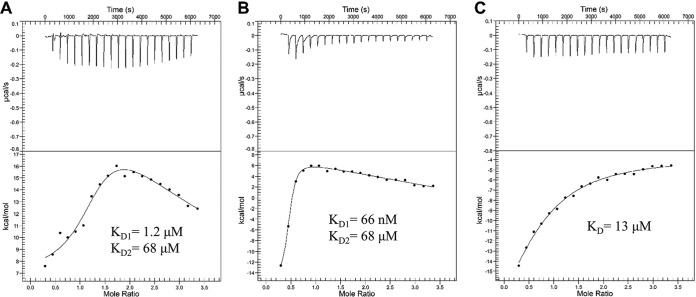
Isothermal titration calorimetry of S1KO, S2KO, and TKO hCP with TdfH. (A and B) The S1KO hCP (A) and S2KO hCP (B) mutants were each titrated into TdfH over 20 injections. Isotherms for both the S1KO and S2KO titrations best fit with a multisite model using NanoAnalyze. (A) Injection of S1KO into TdfH returned affinities (*K_D_*) of 1.2 μM and 68 μM. (B) Injection of S2KO into TdfH returned affinities (*K_D_*) of 66 nM and 68 μM. (C) The isotherm for the TKO titrations showed the best fit to an individual site model. Injection of TKO into TdfH returned an affinity (*K_D_*) of 13 μM. The S1KO, S2KO, and TKO isotherms all had dramatically reduced kcal/mol heat release compared to wild-type hCP.

ITC was employed in order to examine the role that each separate metal-binding site of hCP plays in the interaction with TdfH. TdfH was mixed with either S1KO or S2KO and administered over 20 injections as described above. Isotherms for the S1KO and S2KO were analyzed with NanoAnalyze, and the best fit was to a two-state model. Similarly to the wild-type protein, both the S1KO and the S2KO mutated proteins demonstrated two distinct binding affinities (for S1KO, 1.2 μM and 68 μM [Fig. 7A]; for S2KO, 66 nM and 68 μM [Fig. 7B]). Taken together, these results suggest that knocking out site 1 had a more negative effect on hCP binding than does knocking out site 2. Further, we also titrated in hCP-TKO with TdfH (Fig. 7C). Interestingly, analysis of the TKO data showed a shift from a multisite model to a single-site model with a single, low affinity of 13 μM. These data align well with the cell-based studies presented in the previous section, where we demonstrated that Zn binding in site 1 appears to be more important for supporting growth of the gonococcus (Fig. 6).

## DISCUSSION

The members of the S100 subfamily of EF-hand calcium-binding proteins have been implicated as being among the major transition metal chelators in inflamed tissues (33, 39). Two TdTs produced broadly by pathogenic *Neisseria* spp. have been found to utilize specific S100 proteins as metal sources in order to overcome host nutritional immunity. The meningococcal TdfH homolog, renamed CbpA, was demonstrated to enable interactions between Neisseria meningitidis and CP (24). Jean et al. subsequently demonstrated that N. gonorrhoeae is able to grow on CP and internalize Zn from CP in a TdfH-dependent manner and that production of TdfH enhanced gonococcal survival in neutrophil NETs (23).

Calprotectin is an obligate dimer that preferentially heterodimerizes and is composed of S100 proteins S100A8 and S100A9 (40, 41). Calprotectin has been documented to bind to Zn, Mn, Cu, and Fe, which contributes to its antimicrobial properties against a variety of pathogens (35–38, 42). Calprotectin is also abundant within NETs (31). N. gonorrhoeae is adept at surviving this influx of neutrophils and their toxic effects. N. gonorrhoeae can inhibit the processes of phagocytosis and phagosome maturation and reduce the production of reactive oxygen species through the expression of surface proteins (26). The phase-variable nature of gonococcal lipooligosaccharides (LOS), the production of a thermonuclease, and the presence of TdfH enable gonococcal survival within NETs.

The binding between TdfH and CP had previously been demonstrated only in the context of whole cells (23); thus, it was formally possible that other membrane factors could influence or be responsible for the interaction. The current ITC experiments (Fig. 4; see also Table S1 in the supplemental material) revealed notable dual-mode interactions between TdfH and hCP, with the first being a high-affinity interaction (equilibrium dissociation constant [*K_D_*], 4.0 nM) and the second an interaction with substantially lower affinity (*K_D_*, 35 μM). Interestingly, mCP bound to TdfH also bound in a dual mode but much more weakly, with *K_D_* values of 0.72 μM and 51 μM. CP coordinates Zn atoms at both site 1 and site 2 (43–45). Given that hCP is a heterodimer with two different transition metal binding sites, it is intriguing to speculate that the dual mode of interaction is due to a preference of TdfH for either site 1 or site 2, on opposite sides of the dimer.

N. gonorrhoeae is an obligate human pathogen with a demonstrated species restriction with respect to interaction between host proteins and gonococcal surface proteins, including the TdTs (20, 22, 46). The competition assay (Fig. 2C and D) demonstrated that mCP does not compete with hCP. This could arise because the affinity of the hCP for TdfH is so much stronger than that of mCP (Fig. 5; see also Table S1). Analysis of the amino acid sequences of human and mouse S100A8 and S100A9 found 58% sequence identity for both and 83% and 74% similarity, respectively. The observation that the evolution of hTf has been driven in part by TbpA positive selection may explain the sequence diversity found between human and mouse S100A8 and S100A9 (47); i.e., the evolution of hCP may have occurred through a similar process of positive selection, driven by binding to TdfH.

The two Zn-binding sites of CP are formed at the dimer interface but are nonetheless very different, with noncanonical site 1 containing six histidine residues and canonical site 2 containing three histidine residues and one aspartic acid residue (Fig. 5) (33, 38, 48, 49). Mutations in either metal-binding site 1 (S1KO) or site 2 (S2KO) of hCP were used to test whether these sites contributed equally to the ability of TdfH to interact with CP and whether these sites were utilized equally for Zn piracy (Fig. 6). Growth of N. gonorrhoeae was significantly hindered when Zn binding was abrogated in site 1. When the S1KO was titrated into purified TdfH, an isotherm with minimal heat release was seen, along with relatively weak binding modes. In contrast, the results showed that S2KO resembled the WT-hCP isotherm, with one high-affinity interaction and a second of substantially lower affinity. Moreover, the S2KO mutant was capable of supporting the growth of the gonococcus when provided as the sole Zn source. The differences in affinity and ability to support the growth of the gonococcus suggest that the absence of the noncanonical site (site 1) of hCP significantly hinders TdfH binding and use as a Zn source. Mutation of the canonical site (as in S2KO) had a minimal impact on the overall affinity compared to WT hCP (Fig. 7). Taken together, these data suggest that the region around site 1 represents the primary site of interaction between TdfH and hCP.

**FIG 8:**
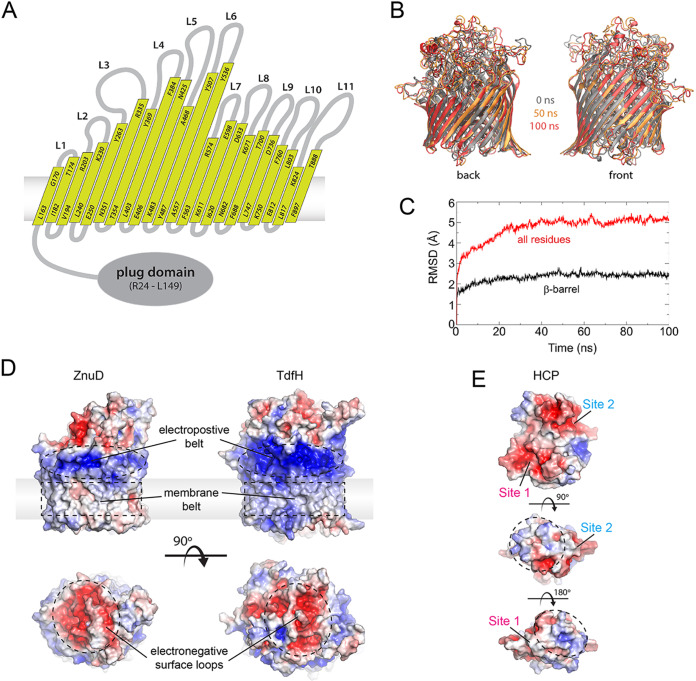
Homology model of TdfH and insight into the interaction with hCP. (A) Membrane topological map of TdfH based on homology modeling using sequence and structural alignments of the closest homologs. (B) A superposition of the TdfH models from the molecular dynamics (MD) simulations at 0, 50, and 100 ns (gray, gold, and red, respectively), showing a stable membrane barrel domain throughout the simulation with little variability. (C) A plot from the MD simulations of the average RMSD of TdfH residues within the barrel only versus all residues. The barrel domain (as well as the plug domain) is significantly more stable than the rest of the protein, which consists mostly of elongated extracellular loops. (D) Electrostatic surface potential comparison of the ZnuD structure (PDB ID 4RDR) with the TdfH homology model. Similar characteristics were observed, including the electropositive belt (dashed ovals), the membrane belt (dashed rectangles), and the electronegative surface loops (bottom, dashed circles). (E) Electrostatic surface potential of human calprotectin (PDB ID 4GGF). Both the site 1 and site 2 regions were observed to be strongly electronegative, while the nonconserved regions highlighted in Fig. 6 were significantly less charged by comparison. A nearly identical result was seen with a model of mouse calprotectin (data not shown), suggesting that electrostatics alone is not responsible for binding to TdfH.

Since there is as yet no TdfH crystal structure available, we used extensive sequence and structural modeling to generate a homology model of TdfH to obtain insight into how it might interact with hCP (Fig. 8). To validate the model, we carried out molecular dynamic simulations, which revealed that the model was stable within a membrane bilayer over the course of the 100-ns simulation. Electrostatic field surface maps of the TdfH model revealed clear domain separation of the membrane belt and properties that align well with those of the previously crystalized meningococcal ZnuD, including an electropositive belt along the surface in proximity to the membrane domain and strongly electronegative surface loops (Fig. 8B). Analysis of the electrostatic field at the surface of hCP showed a predominantly electronegative charge around site 1 and site 2, making it unlikely that those directly participate in binding at the surface loops. Rather, regions encompassing sites 1 and 2 may interact along the electrostatic belt. Interestingly, the divergent ends of hCP that are in close proximity to site 1 and site 2, which we hypothesize may contribute most to the interaction between hCP and TdfH, were not strongly charged (Fig. 8C). Our homology model will enable the design of mutation experiments to interrogate the exact contributions that the loops of TdfH make to the interaction with hCP. Understanding how the TdfH loops contribute to hCP binding will also aid in the performance of studies to produce a better antigen for eventual inclusion into a vaccine for the prevention of N. gonorrhoeae infection, similarly to the use of mutants of TbpB in Haemophilus parasuis to design prophylactic strategies (50).

Our study has provided a detailed view of the elegant interactions between a bacterial transporter and the cognate host ligand. Identification of a species preference for CP informs the need to deploy an hCP transgenic mouse model for further *in vivo* function and immunogenicity testing. Moreover, the finding that site 1 of hCP may be the preferential site of Zn piracy will further guide mutagenesis experiments aimed at deciphering the molecular mechanism used by TdfH to enhance gonococcal virulence.

## MATERIALS AND METHODS

### Neisserial growth conditions.

All strains used in this study are listed and described in Table 1. Neisseria gonorrhoeae was maintained on GC medium base (GCB; Difco) supplemented with Kellogg’s supplement I (51) and 1 μM Fe(NO_3_)_3_ at 37°C in 5% CO_2_. Zinc-restricted growth of N. gonorrhoeae was achieved through inoculation of single colonies grown on GCB medium plates supplemented with 13 μM Fe(NO_3_)_3_ into a Chelex-treated defined medium (CDM) containing 13 μM Fe(NO_3_)_3_. The zinc-specific chelator, *N*,*N*,*N′*,*N′*-tetrakis(2-pyridinylmethyl)-1,2-ethanediamine (TPEN), was added to reach a final concentration of 1 μM at the beginning of the log phase for all cultures to generate a Zn-restricted environment. Cultures were incubated with shaking at 37°C in an atmosphere of 5% CO_2_.

### Cloning, expression, and purification of TdfH.

The full-length *tdfH* gene (Neisseria gonorrhoeae 0952 [NGO0952]) was subjected to codon optimization for expression in E. coli (Bio Basic). The *tdfH* gene was subcloned into the pHIS2 plasmid using NcoI and XhoI restriction sites. Expression into inclusion bodies was performed in BL21(DE3) cells, induced by addition of 0.2 mM isopropyl-d-1-thiogalactopyranoside (IPTG) after growth to an optical density at 600 nm (OD_600_) of ∼1.0 and induction at 37ºC for 3 h. The cells were then harvested and resuspended in 1× PBS pH 7.4 (10 ml per gram of cell paste) supplemented with phenylmethylsulfonyl fluoride (200 μM final concentration) and DNase I (10 μg/ml final concentration). The cell suspension was lysed by three passes through an Emulsiflex C3 homogenizer (Avestin) at 15,000 lb/in^2^. The lysate was centrifuged at 7,000 × *g* for 20 min at 4ºC, and the pellet was washed three times with 1× PBS supplemented with 1% Triton X-100 and 5 mM EDTA (pH 7.4), one time with 3 M urea–1× PBS, and two times with 1× PBS–5 mM EDTA (pH 7.4) using a Dounce homogenizer.

Washed inclusion bodies were resuspended to 5 to 10 mg/ml in 8 M urea containing 2.5 mM β-mercaptoethanol (BME) in a Dounce homogenizer and supplemented with 0.5% Sarkosyl. This was mixed for 15 min at room temperature and then centrifuged for 15 min at 32,000 × *g*. The supernatant was then diluted 60% in refolding buffer (20 mM Tris-HCl [pH 8.0], 200 mM NaCl, 10% glycerol, 0.17% n-dodecyl-β-d-maltoside [DDM]) and dialyzed overnight at 4ºC against a 20× volume of 1× PBS (pH 7.4).

The dialyzed sample was centrifuged at 32,000 × *g* for 15 min at 4ºC and further purified using a linear gradient (25 to 300 mM imidazole) with a Ni-NTA column attached to an AKTA purifier (GE Healthcare) in buffer A (1× PBS [pH 7.4] buffer, 0.05% DDM) and buffer B (1× PBS [pH 7.4] buffer, 0.05% DDM, 1 M imidazole). Peak fractions were verified by SDS-PAGE, and the purest fractions were combined and treated with TEV protease at 4ºC overnight in dialysis into 1× PBS. The sample was then passed over a second Ni-NTA column and the flowthrough concentrated and further purified using a Superdex 200 Increase 10/300 GL column (GE Healthcare) and 1× PBS [pH 7.4] supplemented with 0.05% DDM.

### hCP, hTf, bTf and preparation and metal loading.

Bacterial expression and purification of wild-type and mutant hCP followed previously described protocols (35, 38). The Zn binding site knockout mutants (site 1, S1KO; site 2, S2KO; both sites, TKO) have His-Asn substitutions for the 4 conserved His residues in site 1 and His-Asn substitutions for the 3 conserved His residues plus an Asp-Ser substitution for the conserved Asp residue in site 2. Mouse S100A8 and S100A9 in pQE32 vectors, kind gifts from Claus Heizmann, were reengineered to remove the His tags. The protein was purified following the protocol used for human CP. Briefly, plasmids were transformed in C41 E. coli cells following standard procedures. For each protein, when the OD_600_ reached 0.6, cells were induced at 37°C by the addition of 1 mM IPTG and allowed to grow 4 to 12 h postinduction. Cells were harvested by centrifugation (6.5 krpm, 20 min, 4°C) and resuspended in lysis buffer (50 mM Tris [pH 8.0], 100 mM NaCl, 1 mM EDTA, 1 mM phenylmethylsulfonyl fluoride [PMSF], 0.5 % Triton X-100). Cells were then sonicated (10 min, 50 W, 5 s on/10 s off) and centrifuged at 20,000 rpm for 20 min. The supernatant was discarded, and the pellet was resuspended in lysis buffer and then sonicated and centrifuged as described above. The pellet was then resuspended in a reaction mixture containing 4 M guanidinium-HCl, 50 mM Tris (pH 8.0), 100 mM NaCl, and 10 mM BME. The solution was centrifuged at 20,000 rpm for 20 min and then dialyzed against 20 mM Tris (pH 8.0) and 10 mM BME. The dialysis buffer was changed 3 times over the course of 12 h. The solution was centrifuged, filtered, and loaded onto a SepharoseQ column (GE) (flow rate = 4 ml/min). After loading, the column was washed with 3 column volumes (CV) buffer A (20 mM Tris [pH 8.0], 10 mM BME) and eluted with a gradient (10 CV, 0 to 0.5 M) to buffer B (20 mM Tris [pH 8.0], 1 M NaCl, 10 mM BME). Relevant fractions were pooled, concentrated, and loaded onto a S75 column. Protein was eluted with 1 CV S75 buffer (20 mM Tris [pH 8.0], 100 mM NaCl, 10 mM BME). Relevant fractions were pooled, flash frozen, and stored at –80°C.

Human transferrin (hTf) was prepared as previously described (20, 23). Briefly, lyophilized hTf (Sigma) was dissolved in 40 mM Tris–150 mM NaCl–10 mM NaHCO_3_ at pH 8.4. The hTf mixture was saturated to ∼30% with FeCl_2_ for 1 h and then dialyzed against excess dialysis buffer (40 mM Tris, 150 mM NaCl, 10 mM NaHCO_3_, pH 7.4) to remove any unbound iron. Apo-bovine transferrin (bTf) was prepared as described for hTf except that no FeCl_2_ was added to the solution. CP was maintained in a 20 mM Tris–100 mM NaCl buffer. Zn loading of CP with ZnSO_4_ was completed as described previously (20) at a 2:1 molar ratio of the CP heterodimer to ZnSO_4_, with the exception of the site 1 and site 2 knockouts, which were saturated at a 4:1 molar ratio to ensure that each of the calprotectin preparations reached 25% Zn saturation. WT CP and the site knockouts were dialyzed at 4ºC overnight against 1 liter 20 mM Tris–100 mM NaCl with 50 g Chelex-100 resin added to remove any unbound metals.

### CP-dependent growth of N. gonorrhoeae.

Cells were grown to the log phase in Zn-restricted CDM as described above being diluted to an OD_600_ of ∼0.02 in a microtiter plate containing appropriately diluted mixtures (premixes) of supplements and/or metals (23, 52). The positive-control premix had 5 μM ZnSO_4_ as the sole Zn source and also lacked TPEN. The negative-control premix was devoid of any Zn source but retained TPEN. The final concentrations after dilution of premixes with gonococcal culture were 7.5 μM hTf, 2.5 μM bTf, 10 μM Zn-saturated CP, and 1 μM TPEN. Microtiter plates were incubated for 8 to 12 h in a BioTek Cytation5 plate reader at 36°C with 5% CO_2_. OD_600_ readings were taken every hour over the length of the growth assay. Two-way analysis of variance (ANOVA) was performed with Tukey posttest statistical analysis using Prism 8.1 on 3 independent biological replicates that were tested in technical triplicate (*n* = 9).

### Isothermal titration calorimetry (ITC).

ITC experiments were performed using a Nano ITC microcalorimeter (TA Instruments) at 25ºC with a constant stirring rate at 300 rpm. Samples were buffer exchanged using a Superdex 200 Increase 10/300 GL (GE Healthcare) column into 1× PBS supplemented with 0.05% DDM. TdfH (300 μl at 20 μM) was first placed in the sample cell, and 50 μl of each CP sample (200 μM) was injected in 20 successive injections of 2.5 μl every 300 s. Control experiments were performed in buffer only. The results were analyzed and fitted using the NanoAnalyze software package (TA Instruments) with an initial/active cell volume of 170 μl per the instructions of the manufacturer. Experiments were performed with hCP and mCP in duplicate, while those performed with hCP-S1KO, hCP-S2KO, and hCP-TKO were performed once each.

### Whole-cell dot blot competition assays and total calprotectin binding assays.

Gonococcal strains were passaged on GCB medium plates 2 days before Zn-restricted liquid growth as described above. Cultures were incubated for approximately 4 h after the addition of TPEN at 37°C with 5% CO_2_ before application onto a nitrocellulose membrane in a dot blot apparatus (Whatman). For the dot blot competition assays, the following strains were applied to the membrane: FA1090, MCV661, MCV662, and MCV936 (described in Table 1). Blots with affixed cells were dried overnight before blocking was performed with 5% skim milk–low-salt Tris-buffered saline (LS-TBS; 50 mM Tris, 150 mM NaCl) for 1 h. hCP was conjugated to horseradish peroxidase (hCP-HRP) via the use of an HRP conjugation kit (Abcam) according to the manufacturer’s instructions. Blots were probed with 0.1 μM hCP-HRP alone, with hCP-HRP plus a 5-fold or 10-fold molar excess of unlabeled hCP, or with hCP-HRP plus a 5-fold or 10-fold molar excess of unlabeled mouse calprotectin (hCP) competitor for 1 h. Blots were washed with LS-TBS before being developed with DAB (3′-diaminobenzidine) C/N (–4-chloro-1-naphthol) substrate (Thermo Fisher). The densitometries of triplicate blots were analyzed using Bio-Rad Image Lab after imaging with a Bio-Rad ChemiDoc imaging system. For the total calprotectin binding assays, nitrocellulose membranes containing WT FA1090, MCV661, MCV662, and MCV936 were blocked with 5% skim milk–LS-TBS for 1 h before being incubated with either 0.5 μM hCP or 0.5 μM mCP for 1 h. The blots were washed with LS-TBS–0.1% Tween 20 before being probed with a 1:100 dilution of an anti-S100A9 antibody (clone MA5-12213; Invitrogen) for 1 h. Blots were washed as described above and then probed with a goat anti-rabbit IgG conjugated to HRP secondary antibodies (Bio-Rad) at a 1:3,000 dilution for 1 h. After a final wash step, the blots were developed with a DAB C/N substrate (Thermo Fisher). Blots were imaged with a ChemiDoc imaging system for densitometry analysis quantitated through Image Lab.

### TdfH-CP complex generation and characterization.

To determine whether TdfH would be able to form a complex with hCP or mCP, a codon-optimized gene sequence for TdfH was subcloned into the pHIS2 and pET20b vectors (modified with an N-terminal 10× His tag and TEV protease site) for expression in E. coli. While expression of the native protein by the pET20b vector was only barely observable by Western blotting, we were able to express TdfH into inclusion bodies (pHIS2 vector) with high yields. We refolded and purified TdfH using a Ni-NTA column and did a final purification step into 1× PBS with 0.05% DDM using size exclusion chromatography. TdfH was incubated with either hCP or mCP at a molar ratio of 1:2 for 1 h with rocking at 4°C. The sample was concentrated to ∼500 μl and then passed over a Superdex 200 Increase 10/300 GL column (GE Healthcare) using 1× PBS (pH 7.4) supplemented with 0.05% DDM and an AKTA purifier (GE Healthcare). Peak fractions were visualized using SDS-PAGE to determine the components in the eluted peak, and the data were compared to results of experiments performed with hCP or mCP alone. The presence of the CP protein coeluting with TdfH indicated the formation of a stable complex. For the TdfH/mCP sample, peak fractions were precipitated using trichloroacetic acid (TCA) to increase the amount of sample loaded on the SDS-PAGE gel to enhance a low signal for better visualization.

### Alignment of human and mouse S100A8 and S100A9 protein sequences.

The sequences of human S100A8 (GenBank accession no. AAH05928.1), S100A9 (GenBank accession no. AAH47681.1), mouse S100A8 (GenBank accession no. NP_038678.1), and S100A9 (GenBank accession no. NP_001268781.1) were aligned in a pairwise manner through the use of Geneious and a Blossom 65 matrix. This alignment was then fed into ESPript 3.0 to produce the final alignment, which included the secondary structural elements taken from PDB (PDB identifier [ID] 4GGF). The residues that differed between human and mouse were then mapped to the surface of the human calprotectin structure using PyMOL (Schrödinger), and the final figures were prepared and assembled in Adobe Photoshop and Illustrator.

### Homology modeling of TdfH.

The sequence of TdfH was inputted into the Swiss-Model server to find the best-matching templates. The resulting 50 structures were all TdTs and ranged in sequence identity from 12 to 20%. Sequences from all 50 structures were then aligned, which gave the best matches for identifying the 22 β-strands of the barrel domain of TdfH; the plug domain is well conserved across all TdTs. Once the strands were identified, a pairwise sequence alignment was manually curated with the Fiu receptor (PDB ID 6BPM) to reflect the strand, loop, turn, and plug locations in the TdfH model. The alignment was then fed back into the Swiss Model to form the final homology model for TdfH. To validate our homology model, we performed a 100-ns molecular dynamics simulation using the TdfH model with a bilayer containing an outer leaflet composed of lipopolysaccharide (LPS) based on Neisseria meningitidis (type 1 lipid A and core L1) and an inner leaflet composed of 16:0 (palmitoyl)-16:1 cis-9 (palmitoleoyl) phosphatidylethanolamine (PPPE), 16:0 (palmitoyl)-18:1 cis-11 (vacenoyl) phosphatidylglycerol (PVPG), and 1,1′-palmitoyl-2,2′-vacenoyl cardiolipin (PVCL) in a ratio of 15:4:1 as done previously (53). Explicit TIP3P water model was used, Mg^2+^ and Ca^2+^ ions were included to intercalate LPS phosphates, and K^+^ and Cl^−^ ions were added to bring the solution ion concentration to 150 mM. The system was constructed using CHARMM-GUI (54, 55) and contained 180,000 atoms. NAMD was used with the CHARMM36 force field to simulate the resulting system using a 2-fs time step (56, 57). VMD was used to generate renderings of original and simulated TdfH structures as well as to calculate root mean square deviation (RMSD) values representing the trajectories (58). Importantly, in these simulations, we observed stability within the barrel domain; however, we also observed significant motion within the loops, which were mostly unstructured, although some secondary structure was observed periodically throughout the simulation. Electrostatic fields at the surface of the structures of hCP (PDB ID 4GGF), TdfH, and ZnuD (PDB ID 4RDR) were mapped using the APBS Electrostatics plugin within PyMOL (Schrödinger). The electrostatic field of a model of mCP prepared using the Swiss-Model server was analyzed in a similar manner.
