# Supplementary material for: Molecular Insight into TdfH-Mediated Zinc Piracy from Human Calprotectin by Neisseria gonorrhoeae
Source: mBio. 2020 May 26;11(3):e00949-20. doi: 10.1128/mBio.00949-20 (PMC7251212; doi:10.1128/mBio.00949-20)
Supplement: TABLE S1 [file mBio.00949-20-st001.docx]

**Supplemental Table 1. Summary of isothermal titration calorimetry parameters.**

|  | hCP | mCP | S1KO | S2KO | TKO |
| --- | --- | --- | --- | --- | --- |
| Best-fit Model | Multi-site | Multi-site | Multi-site | Multi-site | Independent |
| Ka_1_ (M^-1^) | 2.5 x 10^8^ ± 4.7 x 10^7^ | 1.4 x 10^6^ ± 3.4 x 10^6^ | 8.6 x 10^5^ ± 3.8 x 10^6^ | 1.5 x 10^7^ ± 1.5 x 10^7^ | - |
| Ka_2_ (M^-1^) | 2.8 x 10^4^ ± 5.9 x 10^3^ | 2.0 x 10^4^ ± 5.6 x 10^4^ | 1.5 x 10^4^ ± 9.2 x 10^4^ | 1.7x 10^4^ ± 1.8 x 10^4^ | - |
| n_1_ | 0.55 ± 0.017 | 0.28 ± 0.063 | 1.3 ± 0.208 | 0.40 ± 0.012 | 0.67 ± 0.12 |
| n_2_ | 1.2 ± 0.5 | 7.8 ± 2.2 | 2.1 ± 6.4 | 8.8 ± 1.0 | - |
| ΔH_1_ (kcal/mol) | -26 ± 1.7 | -50 ± 19 | 3.2 ± 17 | -4.6 ± 5.9 | -25 ± 5.04 |
| ΔH_2_ (kcal/mol) | -32 ± 4.7 | 50 ± 7.4 | 50 ± 20 | 25 ± 13 | - |
| K_D1_ (M) | 4.0 x 10^-9^ | 7.3 x 10^-7^ | 1.2 x 10^-6^ | 6.6 x 10^-8^ | 1.3 x 10^-5^ ± 3.8 x 10^-6^ |
| ΔS_1_ (cal/mol·K) | -50 | -140 | 38 | 17 | -62 |
| K_D2_ (M) | 3.5 x 10^-5^ | 5.1 x 10^-5^ | 6.8 x 10^-5^ | 6.0 x 10^-5^ | - |
| ΔS_2_ (cal/mol·K) | -81 | 190 | 190 | 101 | - |
